# Dr. Mossman, on the Effects of Digitalis in Consumption

**Published:** 1799-08

**Authors:** Geo. Mossman

**Affiliations:** Bradford, Yorkshire


### *To the Editors of the Medical and Physical Journal.*

GENTLEMEN,

THE discussion of a question, involving in its solution a large portion of the fate of mankind, challenges the attention of every one; and surely the ingenious Dr. BEDDOES is entitled to much applause, for the *industry*, which he has exhibited, in his attempts to obviate the fatality of *phthisis*; we ought not, however, to venerate any opinion, though it spring from brilliancy of talent, unless it possess the solid supports of observation and experiment. For several years, I too have attended closely to the origin, progress, and method of affording relief in *phthisis pulmonalis*. My feelings having been almost daily arrested by the melancholy ravages of this disease, I became anxiously solicitous to alleviate its most distressing symptoms. In the prosecution of my inquiries, I have endeavoured to set aside the influence of every preconceived opinion, and to unfetter myself from the tenets of the schools. I can with confidence assert, that my sole ambition has been, to arrive at a knowledge of the truth. I have to lamen much that I have not approached nearer to the object of my pursuit.

It is, however, to be expected, that some additional information upon the subject of *phthisis*, should be the fruit of the now prevailing passion for speculation; and there can be no pretence for saying, that the success of the *old practice* ought to supersede the employment of *new remedies*, in the treatment of a disease, which destroys, annually, *eighty thousand* of the inhabitants of this island. My papers on *phthisis* have long been in a state nearly ready for the press, and I have kept them back, that I might be able to appreciate appreciate the merit of some of the fashionable medicines: the *Digitalis* ranks high in this list.—Upon the supposed action of *Fox-glove*, I shall make no comment; but merely give a brief detail of a few cases, in which I have lately administered it; and if it be consistent with the plan of your undertaking, I have it in contemplation to furnish your repository with additional cases of *phthisis*. In the execution of this design, I shall give a display of those means, which I have found most effectual, in alleviating, *palliatively* or *radically*, the symptoms of the disease.

I am, Gentlemen,

With respect,

BRADFORD, YORKSHIRE, Your's obediently,

*July 9th*, 1799. GEO. MOSSMAN.

### CASE I.

May 19th. J. S. aged 40, previous to his indisposition, was a strong, athletic man. In January last he performed a long journey in a very rainy day, and sat in his wet clothes to a late hour; the following day he was seized with symptoms of *pneumonia*. For a fortnight he refused to have medical aid; at the end of that period, however, he was persuaded to lose some *blood*, and afterwards to take an *emetic*; but without any apparent relief. Since that time he has taken no medicine; he is now in a state of considerable emaciation, and seems approaching to the last stage of *phthisis pulmonalis*. His pulse is about 120; and there is very constantly an exacerbation of fever towards night, accompanied with excessive heat and thirst, till a profuse perspiration taking place about two or three o'clock in the morning, produces a temporary solution of an exquisitely formed *hectic*; colliquative sweats and diarrhœa, alternate with each other. His appetite still remains pretty good, more especially in the morning. His cough is almost incessant, and is attended with a purulent expectoration. He has frequently had a slight hæmorrhage from his lungs, which was very generally followed by a sensation of much soreness in his chest. He can lie down with ease upon his left side, but if he attempt to lie upon his right side, or his back, he is immediately assailed by a sense of suffocation. I enjoined him to live on *milk and eggs exclusively;* and ordered a large *blister* to be applied to the *sternum*. I also prescribed a grain of the *digitalis* to be taken four times a day, with ten drops of the *barytes muriata.*—When his cough was very teizing, he had continual recourse to the use of the *trochisci glycyrrhizæ cum opio*. For several weeks, he scrupulously adhered to the regimen, and to the plan of treatment above recommended, without any *permanent* advantage; the *blister* only, seemed to relieve the *dyspnœa*, and the *troches* his cough. His pulse, in frequency, became subject to great great irregularity; on the ſame day it often beat from 90 to 130 ſtrokes in the minute. From the time of his taking the *digitalis*, he complained of an unuſual heavineſs and drowſineſs; and in ten days afterwards he felt much nauſea, and a tendency to vomit.—Theſe were the only ſtriking phenomena which I could perceive during the exhibition of the medicines already mentioned. The progreſs of the diſeaſe was certainly not arreſted for a ſingle day. He is now confined to his bed, and it is probable, that a very ſhort period, indeed, will terminate his ſufferings. It ought, however, to be obſerved, that the repeated application of *bliſters*, and the occaſional uſe of the *troches*, have very powerfully aſſiſted in ſmoothing the paſſage.

Case II.

May 21ſt. C. G. a ſoldier, aged twenty-one, was ſeized with ſymptoms of *pneumonia*, about three months ago, He was with his regiment, and what remedies were employed to procure relief, I know not; but when he became my patient, he laboured under every ſymptom of a well-marked *phthiſis pulmonalis*. He was enjoined the *ſame regimen*, and had the *ſame remedies*, which are recommended in the preceding caſe; and with effects, almoſt preciſely *ſimilar*;—he died about a week ago.

Case III.

May 25th. M. B. aged thirty, was ſafely delivered of a healthy child, about Chriſtmas laſt, and recovered remarkably well. A few weeks afterwards ſhe was ſeized with ſymptoms of *pneumonia*; for which ſhe took little or no medicine. Her pulſe is 150;—her other ſymptoms are ſtrongly characteriſtic of common caſes of confirmed *phthiſis*. With the exception of *bliſtering*, I preſcribed for her the ſame remedies which I employed in the two foregoing caſes.—A *diarrhœa* occurred previous to the exhibition of any medicine, and continued for a few days, during which, her pulſe ſunk from 150 to 120; but whether from the effects of the *diarrhœa*, or from her medicines, I cannot ſay:—the *digitalis* ſeemed to produce ſickneſs and nauſea. She took it very regularly for three weeks; and at the end of that period, finding no relief, ſhe abandoned the idea of recovery, and refuſed further medical aid. She is ſince dead.

Case IV.

May 25th. M. H. aged twenty-two, was ſeized with a cough, dyſpnœa, and pain in the left ſide, accompanied with a conſiderable degree of fever. She was then in the fifth month of pregnancy, and employed no means to obtain relief. The ſymptoms thus neglected, continued to increaſe, till every ſymptom of a well-marked *phthiſis* was ſufficiently apparent. On the 29th of April ſhe was ſafely delivered of a healthy child.

During

During the firft fortnight fhe appeared to do extremely well; but at the end of that period, every *phthifical* fymptom recurred with additional violence. I prefcribed as in cafe the firft. She expreffed a very ftrong defire to live, and for feveral weeks, with much fortitude and much exactnefs, fhe fcrupuloufly adhered to the plan recommended to her; but, except that her cough was in fome meafure relieved by the *troches*, fhe found no perceptible effect from her medicines.—She is ftill living.

### Case V.

June 14th. J. M. aged nineteen, of a *phthifical* habit, has been for two years paft, occafionally afflicted with a bad cough, accompanied by a purulent expectoration. After raifing a heavy weight yefterday, he was fuddenly feized with a vomiting of blood. He continued, at intervals, during the evening and night, to difcharge blood, fometimes in the form of dark-coloured *coagula*, and fometimes of the appearance of *pure arterial* blood. I faw him to-day at noon, he complained of extreme weaknefs, of forenefs in his cheft, and his breathing was rather difficult; his pulfe was feeble, and 140. It was not eafy to afcertain the quantity of blood loft, but it muft have been from two to four pounds. I prefcribed the *digitalis*, in the dofe of a grain every two hours, followed by a weak folution of *magnefia vitriolata*. After he had taken a few dofes of the medicine, the hæmorrhage returned no more. When I faw him laft, (a week after the attack) he appeared to be in a ftate of convalefcence.

(To be continued.)

